# The 6-months follow-up of the TREAT-CAD trial: Aspirin versus anticoagulation for stroke prevention in patients with cervical artery dissection

**DOI:** 10.1177/23969873251315362

**Published:** 2025-02-05

**Authors:** Stefan T Engelter, Lukas S Enz, Flavia Ravanelli, Josefin E Kaufmann, Henrik Gensicke, Sabine Schaedelin, Andreas R Luft, Christoph Globas, Barbara Goeggel-Simonetti, Urs Fischer, Davide Strambo, Georg Kägi, Krassen Nedeltchev, Timo Kahles, Lars Kellert, Sverre Rosenbaum, Regina von Rennenberg, Alex Brehm, David Seiffge, Susanne Renaud, Tobias Brandt, Hakan Sarikaya, Annaelle Zietz, Johannes Wischmann, Alexandros A Polymeris, Sandro Fischer, Leo H Bonati, Gian Marco De Marchis, Nils Peters, Christian H Nolte, Hanne Christensen, Susanne Wegener, Marios-Nikos Psychogios, Marcel Arnold, Philippe Lyrer, Christopher Traenka

**Affiliations:** 1 Department of Neurology and Stroke Center, University Hospital Basel and University of Basel, Basel, Switzerland; 2 Neurology and Neurorehabilitation, University Department of Geriatric Medicine FELIX PLATTER, University of Basel, Basel, Switzerland; 3 Department of Clinical Research, University of Basel, Basel, Switzerland; 4 Division of Vascular Neurology and Neurorehabilitation, Department of Neurology, University Hospital of Zurich and University of Zurich, Zurich, Switzerland; 5 Cereneo, Center for Neurology and Rehabilitation, Vitznau, Switzerland; 6 Center for Stroke Research Berlin (CSB), Charite-Universitätsmedizin Berlin, Berlin, Germany; 7 Department of Neuropediatrics, Institute of Pediatrics of Southern Switzerland, San Giovanni Hospital, Bellinzona; 8 Faculty of Biomedical Sciences, Università della Svizzera Italiana, Lugano, Switzerland; 9 Department of Neurology, University Hospital Bern, University of Bern, Bern, Switzerland; 10 Stroke Center, Service of Neurology, Department of Clinical Neurosciences, University Hospital of Lausanne and University of Lausanne, Lausanne, Switzerland; 11 Department of Neurology and Stroke Center, Cantonal Hospital St.Gallen, St.Gallen, Switzerland; 12 Department of Neurology and Stroke Center, Cantonal Hospital Aarau, Aarau, Switzerland; 13 Department of Neurology, University of Bern, Bern, Switzerland; 14 Department of Neurology, Ludwig Maximilian University (LMU), Munich, Germany; 15 Department of Neurology, Copenhagen University Hospital, Bispebjerg, Copenhagen, Denmark; 16 Department of Neurology and Experimental Neurology, Charite-Universitätsmedizin Berlin, Berlin, Germany; 17 Department of Neuroradiology, University Hospital Basel, Basel, Switzerland; 18 Division of Neurology and Stroke Unit, Neuchâtel Hospital Network, Neuchâtel, Switzerland; 19 Swiss National Accident Insurance Fund SUVA, Lucerne, Switzerland; 20 Research Department, Reha Rheinfelden, Rheinfelden, Switzerland; 21 Berlin Institute of Health at Charite, (BIH), Charite-Universitätsmedizin Berlin, Berlin, Germany

**Keywords:** Cervical artery dissection, stroke in the young, treatment, treatment duration

## Abstract

**Introduction:**

Cervical artery dissection is a major cause of stroke in the young. The optimal choice and duration of antithrombotic treatment for stroke prevention are debated, particularly beyond 3 months after symptom onset.

**Patients and methods:**

TREAT-CAD (**TREAT**ment of **C**ervical **A**rtery **D**issection) was a randomized controlled trial with blinded outcome assessment comparing non-inferiority of aspirin to anticoagulation (Vitamin-K-antagonists) in participants with symptomatic, Magnetic-Resonance-(MR)-imaging-verified cervical artery dissection. TREAT-CAD could not establish non-inferiority of aspirin to anticoagulation at 3 months. Thereafter participants could continue antithrombotic medication and obtained a standardized assessment of clinical and MR-Imaging outcomes between 3 and 6 months. As crossover to the other treatment arm was possible, we performed an as-treated analysis as main analysis. The main outcomes were new clinical (ischemic stroke, intracranial/major extracranial bleeding, or death) and new MR-Imaging outcomes (ischemic or hemorrhagic brain lesions).

**Results:**

Among the 122 participants in the as-treated analysis, 3/93 (3.2%) aspirin-treated participants had new clinical (*n* = 1) and MRI-outcomes (*n* = 2) between 3 and 6 months while 1/29 (3.4%) anticoagulated participants had an MRI-outcome (*n* = 1). All outcome events were hemorrhagic while ischemic events were absent. No deaths occurred. This yields an absolute difference of 0.2% (95% CI −8.0% to 7.5%, *p* = 1.0).

**Discussion and conclusion:**

During the extended follow-up period of a controlled randomized trial comparing aspirin to anticoagulation in cervical artery dissection, outcomes between 3 and 6 months after randomization occurred rarely, similarly often in both groups and were exclusively hemorrhagic events. Thus, studies balancing benefits versus harms of antithrombotic treatment beyond 3 months are warranted. Registration: ClinicalTrials.gov: NCT02046460. https://clinicaltrials.gov/ct2/show/NCT02046460.

## Graphical abstract

**Graphical abstract:**
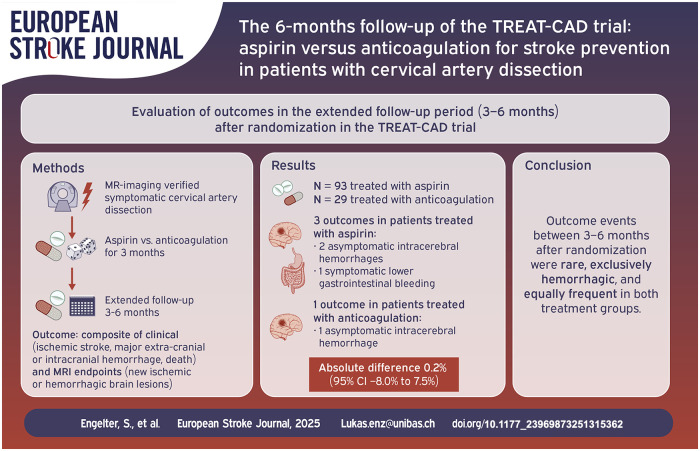

## Introduction

Cervical artery dissection (CeAD) is a major cause of stroke in young individuals.^1–5^ For stroke prevention, either antiplatelets or anticoagulants are used.^6,7^ Two randomized controlled trials (RCTs) – CADISS^8,9^ and TREAT-CAD^10,11^ – compared early antiplatelet with anticoagulation therapy in CeAD. None of the trials nor a meta-analysis across both trials showed superiority of either approach.^12^ Additionally, optimal duration of antithrombotic treatment is unknown. Current guidelines recommend continuing antithrombotic treatment for at least 3–6^13^ or even 6–12 months^14^ based on expert opinions. Both CADISS and TREAT-CAD evaluated primary outcomes after 3 months of randomly allocated treatment, while there are few data on the occurrence of ischemic or hemorrhagic events beyond 3 months after CeAD.^9^

In TREAT-CAD, which in contrast to CADISS used clinical and MR-imaging outcome, participants could continue on antithrombotic medication and obtained a standardized assessment on the occurrence of new clinical and MRI imaging outcomes at 6 months as foreseen in the protocol.^11^ These data allow to compare benefits and harms of aspirin versus anticoagulants in the extended follow-up period from 3 until 6 months after CeAD.

In detail, we report on the frequency of both clinical and MR-imaging outcomes between 3 and 6 months after CeAD among the per-protocol participants of the TREAT-CAD trial, in whom follow-up until 6 months was available. Comparisons include type of treatment (aspirin vs anticoagulant) and type of outcome event (ischemic and hemorrhagic).

## Methods

TREAT-CAD (Biomarkers and antithrombotic treatment in cervical artery dissection) was a multicenter, open-label, randomized controlled non-inferiority trial comparing aspirin to anticoagulation with vitamin K antagonists (allocation ratio 1:1) in the treatment of CeAD with regard to the occurrence of a composite of clinical outcomes (stroke, major hemorrhage, death) and MRI outcomes (new ischemic or hemorrhagic brain lesions).^11^ The trial design, sample size calculations,^11^ and randomization and blinding procedures have been published in detail.^10^ The primary endpoint was assessed in the per-protocol population at the end of the 3-month interventional period as reported previously.^10^ In brief, among the 173 participants of the per-protocol population, study treatment was aspirin in 91 (53%) and anticoagulation in 82 (47%) participants and was started at a median of 3 days after hospital admission. Until the 3 months follow-up visit, clinical or MRI outcomes had occurred in 21 (23%) patients in the aspirin group and in 12 (15%) in the anticoagulation group.^10^

According to the TREAT-CAD protocol^11^ participants could be followed up until an optional 6-month follow-up-visit during which the same standardized clinical and MRI assessments as in the 14 day-and/or 3 month visit were applied. We performed a pre-specified 6-month analysis of the per-protocol-participants of the TREAT-CAD trial for whom outcome data at 6 months were available.

As crossover to the other treatment group at 3 months was possible, our primary analysis was an as-treated analysis including all participants with (i) clinical and MRI data available at 6-months follow-up and for whom (ii) detailed information about the antithrombotic treatment regimen taken between 3 and 6 months was available. Participants who had switched to antithrombotic treatment regimen other than specified in the protocol (e.g. to a direct oral anticoagulant) or for whom the type of antithrombotic treatment was unknown and those who were not interested in participating in follow-up after 3 months were excluded from the analysis.

**Figure 1.**
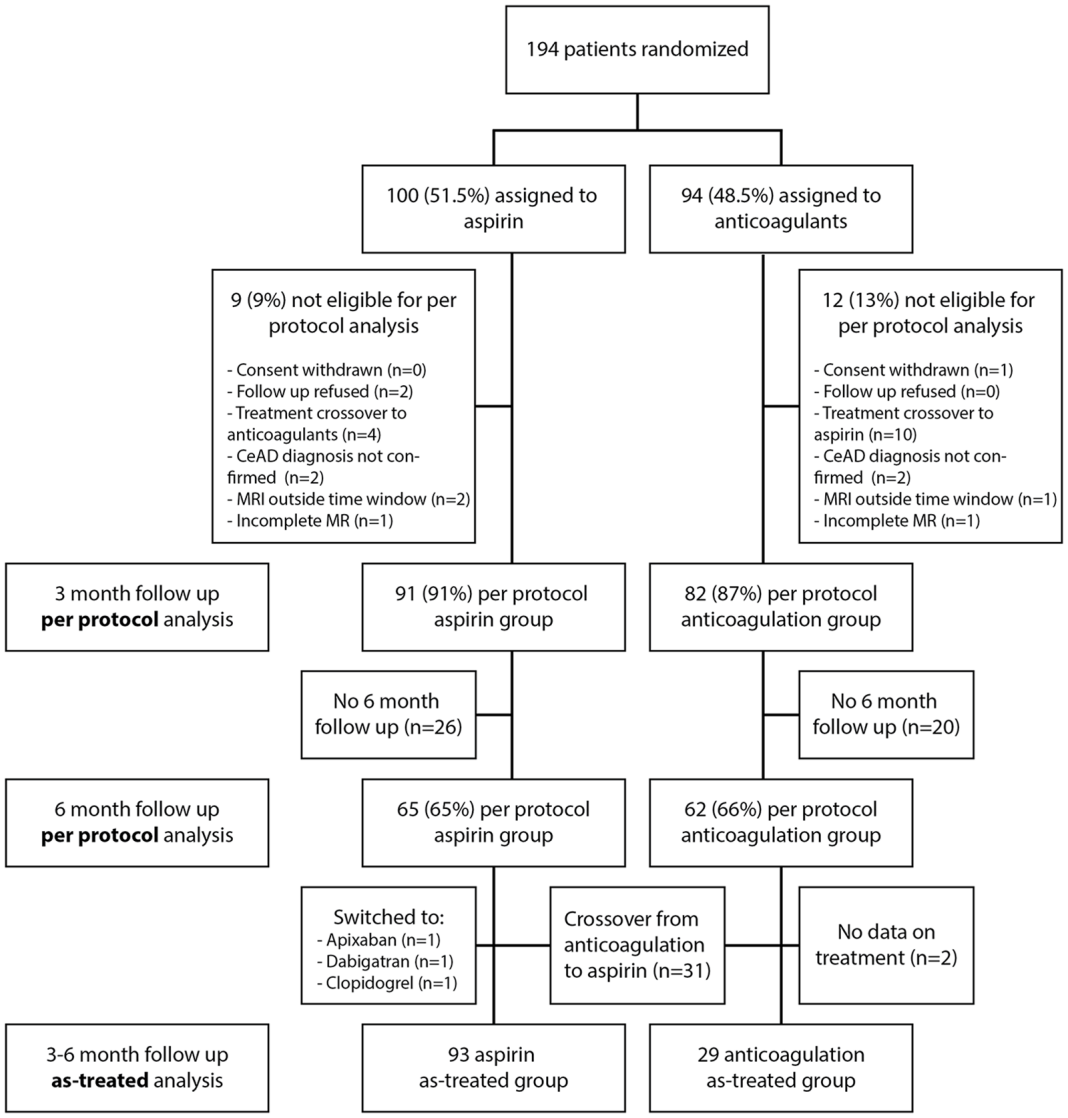
Trial flow chart. The 3 month per-protocol analysis has been reported previously.^10^

The trial flow chart is shown in Figure 1. As sensitivity analysis, we analyzed the occurrence of outcome events at 6 months stratified to the type of antithrombotic medication participants had initially taken, irrespectively of any crossovers (6-month-follow-up of the original per-protocol population).

### Standard protocol approvals, registrations, and patient consents

The trial was approved by the relevant ethics committees and regulatory authorities for each center in Switzerland, Germany, and Denmark. Written consent was obtained from all study participants. The TREAT-CAD trial was registered at clinicaltrial.gov (NCT02046460, https://clinicaltrials.gov/ct2/show/NCT02046460, registration 23.1.2014, first patient inclusion 3.10.2013). We used the CONSORT reporting guidelines. The research plan and statistical analysis plan are available in the Supplements.

### Participants

Participants included in this pre-planned secondary analysis of TREAT-CAD met the following criteria: they had had a MR-verified clinically symptomatic cervical artery dissection, adhered to allocated study medication for the interventional phase of 3 months, had completed the 3-month assessment and had agreed to an extended follow-up including assessments for clinical and MRI outcomes at 6 months.^10,11^

### Intervention

During the extended follow-up period of TREAT-CAD, participants received either aspirin (75 mg (Center in Copenhagen) or 100 mg (all other centers) per day) or anticoagulation with vitamin-K-antagonists (phenprocoumon [Marcumar™], or acenocoumarol [Sintrom™], or warfarin [Marevan™]) for the period between 3 and 6 months after the index event.

### Outcomes

The main outcome of this 6-month data analysis was a composite of clinical (ischemic stroke, major extracranial or intracranial hemorrhage, death) and MRI outcomes (new ischemic or hemorrhagic brain lesions) between 3 and 6 months, applying criteria identical to those used in prior analyses.^10,11^ Detailed definitions of the outcomes are listed in the Supplemental Appendix. Clinical outcomes were independently adjudicated by an independent Clinical Event Adjudication Committee, as done in the previous publication. The members of the committee (SR and TB) received clinical source data and therefore were aware of the treatment allocation.

MRI outcomes occurring between 3 and 6 months after randomization were adjudicated by the consensus of two independent readers (FR and ChT), with a third reader (STE) involved in case of disagreement between first and second reader – as done in prior research.^15^ Readers were blinded to the allocated treatment and the clinical outcome of participants.

### Follow-up assessments

Participants were followed up three times: (i) clinically and with MRI at 14 (±10) days after enrollment (ii) clinically at 90 ± 30 days, (iii) clinically and with MRI at 180 ± 30 days. MRI assessment included the following MRI-sequences: (i) Diffusion Weighted Imaging (DWI) including Apparent Diffusion Coefficient (ADC) maps to detect new acute ischemic brain lesions, (ii) paramagnetic sequences, that is, T2*w gradient echo (GRE) or Susceptibility Weighted Images (SWI) to detect new hemorrhagic brain lesions.^10,11^ For the current analysis we identified all clinical or MR outcomes, which were new at 6 months, that is, they were not present at prior follow-up assessments which were performed at 14 and 90 days (clinical outcomes) and at 14 days respectively (in case of MRI outcomes).

### Statistical analysis

We compared the occurrence of the primary endpoint across both treatment groups between 3 and 6 months. The type of antithrombotic treatment (i.e. aspirin or anticoagulation) that patients had taken between 3 and 6 months or from 3-month until the occurrence of an outcome event defined the treatment group for the as-treated analysis.

As sensitivity analysis we repeated the analysis but this time, the type of antithrombotic treatment, participants had received in the interventional 3-month phase, defined the treatment group also for the 3–6-month analysis irrespective of any treatment changes in the observational phase between 3 and 6 months. This approach corresponds to the report about the final 12 months results of the CADISS trial.^9^

We assessed the absolute risk difference between participants in the aspirin and anticoagulation groups with its 95% confidence interval using Wilson`s method (continuity-corrected modification of Wilson`s score method). Continuous data are reported by mean and standard deviation. For categorical variables, absolute and relative frequencies are presented. The analyses were conducted using the software package R.

### Handling of missing data – sensitivity analysis

Missing data regarding type of antithrombotic taken between 3 and 6 months were not imputed. As the sample size for the extended follow-up-period was determined by the randomized phase from 0 to 3 months, no prior power calculations had been done for the extended follow-up between 3 and 6 months. We instead – post-hoc – estimated the number of additional outcomes required to exclude zero from the 95% confidence interval of the main analysis (i.e. to demonstrate superiority of one over the other comparator treatment option). Additionally, we performed a sensitivity analysis using the type of antithrombotic treatment that participants had received in the first 3-month phase, as originally randomized (per-protocol analysis).

### Handling of treatment cross-over

At the end of the interventional phase at 3 months, crossover to the other treatment group was possible. Therefore, we performed the as-treated analysis as the main analysis, which compared the occurrence of clinical or MRI outcomes according to the type of antithrombotic treatment participants had actually received between 3 and 6 months.

### Independent data access and analysis

The three first and the last author had full access to all the data in the study and take responsibility for its integrity and the data analysis.

### Data availability

Datasets generated or analyzed within the present study can be made available from the corresponding author upon reasonable request.

## Results

### Participants and interventions

In 125 of the 127 (98%) participants of the TREAT-CAD per-protocol population, for whom data on outcomes between 3 and 6 months were available, detailed information about the type of antithrombotic medication taken between 3 and 6 months was available. Two participants were excluded because their antithrombotic treatment regimen taken between 3 and 6 months was unknown. None of these had outcome events 3–6 months after randomization. In 122 of 125 (98%) participants, either aspirin or a vitamin-K-antagonist were used – that is, one of the two comparator treatment options in TREAT-CAD. Three additional participants had been switched to a treatment not mentioned in the TREAT-CAD-protocol at the 3-month-visit and were excluded from the primary analysis. Initially, all three had been randomized to, allocated to and taken aspirin for 3 months. At the 3-month-visit they were switched from aspirin to apixaban (*n* = 1), dabigatran (*n* = 1), or clopidogrel (*n* = 1), for reasons not known. None had reported outcome events between 3 and 6 months.

The remaining 122 participants comprised the study population for the as-treated analysis.

Among the as-treated-study participants, 93 (76%) took aspirin and 29 (24%) took vitamin-K antagonists between 3 and 6 months. Among aspirin takers, 31/93 had crossed over from the vitamin-K antagonists group to aspirin at 3 months, while 62 took aspirin since randomization. Ninety-one of the 93 participants taking aspirin during the extended follow-up period, received a daily dosage of 100 mg, while two participants received 75 mg aspirin per day. Among participants taking vitamin-K antagonists between 3 and 6 months, all took vitamin-K antagonists already since randomization and none had crossed from the aspirin to the vitamin-K antagonists group at 3 months.

Mean age was 46.6 years (SD 11.1), 80 (65.5%) patients were male and 76 (62.3%) presented with ischemic events (with or without accompanying local symptoms at baseline; i.e. 61 with acute ischemic strokes including 4 retinal infarcts, 15 TIAs including 3 with amaurosis fugax, and 5 had multiple ischemic events). Purely local symptoms (cervical pain, headache, cranial nerve palsy, Horner syndrome, tinnitus) were reported in 46 (37.7%) patients. The carotid artery was dissected in 81 (66.4%) participants and the vertebral artery in 42 (34.4%) participants, including 5 (4.1%) patients with simultaneous involvement of multiple arteries. Intramural hematoma was the most frequent imaging characteristic identified in baseline MR imaging in 118 (96.7%) patients.

Baseline characteristics are summarized in Table 1 and were comparable in both groups.

**Table 1.** Baseline characteristics.

Characteristic	Per-protocol population with 6 month-follow up-data (*n* = 127)			Missing follow up at 6 months from 3 month per-protocol population (*n* = 46)
	As treated population 3–6 months (Aspirin vs Anticoagulation (*n* = 122)	Unknown treatment after 3 months or non (*n* = 5)		Original randomization: *n* = 26 Aspirin *n* = 20 VKA
	Aspirin (*n* = 93)	VKA. (*n* = 29)		
Age (years, mean (SD))	45.3 (10.4)	51 (12.1)	44.2 (16.4)	45 (9.9)
Male sex, *n* (%)	59 (63.4)	21 (72.4)	2 (40)	28 (60.9)
Site of dissection				
Internal Carotid Artery, *n* (%)	65 (69.9)	16 (55.2)	3 (60)	31 (67.4)
Vertebral Artery, *n* (%)	29 (31.2)	13 (44.8)	2 (40)	17 (37)
Multivessel dissection, *n* (%)	3 (3.2)	2 (6.9)	1 (20)	7 (15.2)
Occlusion of dissected artery, *n* (%)	29 (31.2)	11 (37.9)	3 (60)	12 (26.7)
Mural hematoma, *n* (%)	90 (96.8)	28 (96.6)	4 (80)	43 (93.5)
Presenting signs/symptoms				
Cerebral ischemic events^a^				
Ischemic stroke	47 (50.5)	12 (41.4)	4 (80)	27 (58.7)
Transient ischemic attack	11 (11.8)	4 (13.8)	1 (20)	6 (13)
Retinal infarct	4 (4.3)	0 (0)	0 (0)	0 (0)
Amaurosis fugax	1 (1.1)	2 (6.9)	0 (0)	4 (8.7)
Local signs				
Cervical pain	48 (51.6)	15 (51.7)	3 (60)	21 (45.6)
Headache	63 (67.7)	21 (72.4)	5 (100)	30 (65.2)
Cranial nerve palsy	12 (12.9)	2 (6.9)	2 (40)	2 (4.3)
Horner’s syndrome	35 (37.6)	11 (37.9)	2 (40)	12 (26.1)
Tinnitus	13 (14)	0 (0)	1 (20)	3 (6.5)
NIHSS score baseline, mean (SD)	1.1 (2.4)	1.2 (2.4)	0.4 (0.5)	1.9 (3.9)
Risk factors				
Hypertension	30 (32.3)	16 (55.2)	2 (40)	7 (15.2)
Hypercholesterolemia	16 (17.2)	7 (24.1)	2 (40)	11 (23.9)
Diabetes	1 (1.1)	2 (6.9)	0 (0)	1 (2.2)
History of smoking	51 (54.8)	18 (62.1)	0 (0)	16 (34.8)
Migraine with aura	15 (16.1)	2 (6.9)	0 (0)	6 (13)
Migraine without aura	13 (14)	3 (10.3)	2 (40)	6 (13)
Mechanical trigger event within 4 weeks prior to enrolment	14 (15.1)	6 (20.7)	1 (20)	7 (15.2)
Infection within 4 weeks prior to enrolment	27 (29)	5 (17.2)	0 (0)	7 (15.2)
Outcome events 0–3 months	15 (16.1)	4 (13.8)	2 (40)	12 (26.1)
Clinical ischemic stroke including retinal infarct	4	0	2	1
Major extracranial hemorrhage	1	0	0	0
MRI ischemic lesions	6	3	2	7
MRI hemorrhagic lesion	7	1	0	8

a Five patients presented with multiple cerebral ischemic events.

### Outcomes stratified to type of treatment

Four of 122 (3.3%) participants had outcomes between 3 and 6 months. Among participants treated with aspirin between 3 and 6 months, 3/93 (3.2%) experienced outcomes: one participant had a major clinical extracranial hemorrhage on day 189 (a lower gastrointestinal bleeding) and two participants had new hemorrhagic MR lesions, not present at prior MR-imaging. In the vitamin-K antagonists group, 1/29 participant (3.4%) experienced a new hemorrhagic MR-imaging lesion, not present at prior MR imaging. The newly detected hemorrhagic MR-lesions met the criteria for microbleeds (Supplemental Figure e-1). In the vitamin-K antagonists group there were no clinical outcome events between 3 and 6 months (Tables 2 and 3). The absolute risk difference between the two groups was 0.2% (95% CI −8.0% to 7.5%, *p* = 1).

**Table 2.** Characteristics of participants with outcome events between 3 and 6 months stratified to the type of antithrombotic treatment taken (as treated analysis).

Patient characteristic	Aspirin^a^			Vitamin-K antagonists^a^
Age	44	55	50	34
Sex	Male	Male	Male	Female
Type of presenting symptoms	Ischemic stroke	Ischemic stroke	Ischemic stroke	Ischemic stroke
Dissected artery	Carotid	Vertebral	Carotid	Vertebral bilateral
Treatment 0–3 months	Aspirin	Aspirin	Vitamin-K antagonists	Vitamin-K antagonists
Treatment 3–6 months	Aspirin	Aspirin	Aspirin	Vitamin-K antagonists
3-month outcome event	hemorrhagic MR lesion	none	Lower gastrointestinal bleeding at day 7	none
Reason for crossover at 3 months	-	-	Lower gastrointestinal bleeding.	-
Outcome event between 3 and 6 months	Recurrent hemorrhagic MR lesion	Hemorrhagic MR lesion	Recurrent lower gastrointestinal bleeding at day 189.	Hemorrhagic MR lesion
Accompanying clinical symptom to MR outcome	none	none	Not applicable	none

a Type of antithrombotic treatment taken in- between 3 and 6 months after randomization.

**Table 3.** Clinical and MR outcomes between 3 and 6 months including outcomes between 0 and 3 months (the latter from Engelter 2021).

Clinical and MRI-outcomes	Per protocol				As treated	
	0–3 month (*n* = 173)		3–6 month (*n* = 127)		3–6 month (*n* = 122)	
	Aspirin (*n* = 91) (%)	VKA (*n* = 82) (%)	Aspirin (*n* = 65) (%)	VKA (*n* = 62) (%)	Aspirin (*n* = 93) (%)	VKA (*n* = 29) (%)
Primary endpoints total	21 (23.1)	12 (14.6)	2 (3.1)	2 (3.2)	3 (3.2)	1 (3.4)
Components of the composite primary endpoint						
Clinical outcomes (all)	7 (7.7)	1 (1.2)	0	1 (1.6)	1 (1.1)	0
− Ischemic stroke	7 (7.7)	0	0	0	0	0
− Major extracranial hemorrhage	0	1 (1.2)	0	1 (1.6)	1 (1.1)	0
− Symptomatic intracranial hemorrhage	0	0	0	0	0	0
− Death	0	0	0	0	0	0
MRI-outcomes (all)	20 (21.9)	11 (13.4)	2 (3.1)	1 (1.6)	2 (2.2)	1 (3.4)
− New acute ischemic brain lesions	9 (9.9)	6 (7.3)	0	0	0	0
− New hemorrhagic brain lesion	9 (9.9)	4 (4.9)	2 (3.1)	1 (1.6)	2 (2.2)	1 (3.4)
− New acute ischemic and new hemorrhagic lesions	2 (2.2)	1 (1.2)	0	0	0	0
MRI-outcomes without symptoms	14 (15.4)	11 (13.4)	2 (3.1)	1 (1.6)	2 (2.2)	1 (3.4)
− New acute ischemic brain lesions	3 (3.3)	6 (7.3)	0	0	0	0
− New hemorrhagic brain lesion	9 (9.9)	4 (4.9)	2 (3.1)	1 (1.6)	2 (2.2)	1 (3.4)
− New acute ischemic and new hemorrhagic lesions	2 (2.2)	1 (1.2)	0	0	0	0

Interestingly, two of the four participants with new endpoints at 6 months had already had the same kind of hemorrhagic outcomes within the first 3 months. First, the participant with a new hemorrhagic MR-imaging lesion under aspirin at 6 months had already had a hemorrhagic lesion first visible (at a different location) within the 3-month assessment period, also under aspirin. Second, the participant who had a gastrointestinal (GI)-bleed at 6 months under aspirin, was the very same participant, who had a GI bleed already within 3 months – under vitamin-K antagonists.

Table 3 summarizes the characteristics of the four participants with new outcome events between 3 and 6 months.

### Sensitivity analysis

Post-hoc, we calculated that for the 95% confidence interval to exclude zero in either direction (i.e. to demonstrate superiority of either treatment over the other), an additional four outcomes among 29 VKA-treated patients would have been necessary (thus 5/29, 17.2%, rather than 1/29 (3.4%)) to show superiority of aspirin. Alternatively, in the opposite direction, an additional 17 outcomes in the aspirin group (thus 20/93, 21.5%, rather than 3/93 (3.2%)) would have been necessary to demonstrate superiority of the vitamin-K antagonists.

The 6-month per protocol population consisted of 127 patients for whom data on outcomes between 3 and 6 months were available (irrespective of any cross-over to the other treatment arm). Of these, 65 (51.1%) had originally been randomized to aspirin and 62 (48.8%) to vitamin-K antagonists (Figure 1). There were four participants with new outcome events between 3 and 6 months, two – both with hemorrhagic MR-outcomes – in the aspirin group (2/65, 3.1%) and two participants – one with a GI-Bleed and one with a new hemorrhagic MR-outcome in the vitamin-K antagonists group (2/62, 3.2%, Table 2), amounting to an absolute risk difference of 0.15% (95% CI −6.1% to 6.4%, *p* = 1). The occurrence of clinical and MR-outcomes at 3–6 months – including those occurring between 0 and 3 months stratified to the treatment groups are summarized in Table 3.

## Discussion

The key findings of the 6-month-follow-up data of the randomized-controlled TREAT-CAD Trial were: First, outcomes between 3 and 6 month after randomization were rare and occurred similarly often under aspirin as under vitamin-K antagonists. Second, all outcome events between 3 and 6 months were hemorrhagic while ischemic events were absent. Third, all hemorrhagic brain MRI lesions were asymptomatic.

Until 3 months after randomization ischemic stroke, major bleeds, or their MRI surrogates had occurred in every fourth-to-fifth TREAT-CAD participant treated with aspirin (23%) but in only every seventh patient in the VKA group (15%).^10^ Though this difference did not reach statistical significance, a possible benefit of vitamin-K antagonists might be present. Our findings indicate that this possible benefit – if present – may possibly be restricted to the acute phase, as any difference between treatment groups regarding the occurrence of new outcome events had disappeared in the period of 3–6 month after randomization. The assumed absence of a benefit of either treatment beyond 3 months, is also supported by the observation that during the extended (i.e. >3 months) follow-up-period in CADISS, there was one stroke each in the vitamin-K antagonists and the antiplatelet group (plus one death among a patient with stroke under antiplatelets).^9^

Prior research^8,10,17^ – recently confirmed^16^ – had shown, that within the first 3 months, ischemic outcomes largely outnumbered hemorrhagic outcomes. Furthermore, the risk of ischemic stroke is highest in the beginning and seems to decline over time.^17^

In line with prior randomized controlled (CADISS)^9^ and observational (STOP-CAD)^16^ studies (Supplemental Table e-1), outcome events in TREAT-CAD occurred early and were predominantly ischemic. The observation of the current study suggesting that hemorrhagic outcomes may dominate the period beyond 3 months has not been reported by the aforementioned studies^9,16^ is therefore novel, should be considered hypothesis-generating and requires confirmation.

In this context, it is of importance, that all outcomes observed between 3 and 6 months after randomization in TREAT-CAD were hemorrhagic, while ischemic outcomes were absent. If these findings are supported by future research, this would not support current recommendations to continue antithrombotic treatment for at least 6–12 months after CeAD.^14^ Instead, future research addressing the risk-benefit-ratio of longer-term antithrombotic treatment in CeAD is required. In this context, it is of importance that in our trial all hemorrhagic brain MRI lesions between 3 and 6 months were asymptomatic.

Interestingly, two of the four participants with new outcome events between 3 and 6 months had had the same kind of – hemorrhagic – outcomes already within the first 3 months. Although spurious coincidences are possible, these observations might suggest the presence of patient-inherent factors which might have contributed to the adverse effect of the antithrombotic medication.

As strengths, all TREAT-CAD centers have expertise in diagnosis and treatment of CeAD, resulting in a high rate of participants who agreed in the optional 6-month follow-up with completed and standardized clinical and MR-imaging assessment. Furthermore, central adjudication and multi-rater assessments of clinical and imaging outcomes increased the validity of the results. Moreover, the analyses reported here were based on data from an RCT, which combined clinical and MRI outcome for an extended follow-up period of up to 6 months. More importantly, clinical and MRI outcomes seemed concordant, which renders spurious findings less likely.

However, we are aware of important limitations. The sample size of our study population disallowed to aim at statistically significant differences regarding the frequency of outcome events between treatment groups. Moreover, the sample size for the extended-follow-up analysis was pre-determined by the randomized-controlled phase of TREAT-CAD. Therefore, for demonstrating – nevertheless – superiority of one (or the other) comparator treatment options, the frequency of outcome events in between 3 and 6 months after randomization must have had been substantially higher than observed – that is, at least four to six times higher as indicated by our post-hoc calculations. Furthermore, the observation that a substantial number of participants crossed over from the vitamin K antagonists group to the aspirin group (while none did in the opposite direction) indicates the presence of an allocation bias due to preferences of participants and treating physicians. The observations, that (i) the per-protocol-analysis and the as-treated-analysis yielded concordant results and (ii) that among participants with outcome events, only one had switched treatment groups, suggested that cross-overs did not relevantly confound our key findings. In addition, for several participants, 6-month outcome data was missing which may have introduced a selection bias.

The use of DWI sequences as means to detect ischemic brain lesions which had newly occurred between 14 days and 6 months follow-up visit involved the risk of missing some lesions (as no follow-up MRI had been performed at the 3-month follow-up visit). This considers in particular ischemic lesions occurring relatively early after the 14-day assessment, as DWI lesions are known to fade out over time,^15,18^ while hemorrhagic lesions are known to persist longer in SWI. This may bias the outcome events toward hemorrhagic events, as ischemic events are more likely to have already faded until the 6-month follow-up assessment. Therefore, it is also possible, that hemorrhagic events among patients treated with aspirin at 3–6 months could have occurred also under the treatment with VKA 14 days–3 months (or vice versa), which could have biased our imaging assessment at 6 months. Moreover, the interpretation of small paramagnetic MRI lesions as solely hemorrhagic brain lesions might be overly simplistic. Some of these lesions may reflect hemorrhagic transformation of a primarily ischemic lesion.^19^ Further, the clinical meaning of such lesions without accompanying clinical symptoms is unclear, taking into account that they can occur after thrombolysis in stroke patients^20,21^ and also in the normal population.^22^ Lastly, recanalization of initially occluded dissected arteries was not routinely assessed during follow-up and could not be accounted for as potential confounder of patient outcome. Considering these limitations, we urge toward a cautious interpretation of our key findings.

Future trials on antithrombotic treatment in CeAD may consider focusing on stroke preventive intervention during the first days to weeks, when the risk of ischemic stroke seems highest.^9,10,17^ In addition, future trials may consider novel developments not available in TREAT-CAD such as the use of direct oral anticoagulants and dual antiplatelet therapy, taking into account their favorable benefit-harm ratio at least on other indications. Thereafter, the clinical usefulness of continuing versus stopping antithrombotic treatment might be worth testing. Indeed, a large observational CeAD cohort study (STOP-CAD) comparing outcomes with different antithrombotic treatment options indicated that benefits and harms of different antithrombotic regimens might differ over time.^16^ However, unbiased data on the optimal duration of antithrombotic treatments balancing their benefits and risks requires large and well powered randomized controlled trials.

## Supplemental Material

sj-docx-1-eso-10.1177_23969873251315362 – Supplemental material for The 6-months follow-up of the TREAT-CAD trial: Aspirin versus anticoagulation for stroke prevention in patients with cervical artery dissection Supplemental material, sj-docx-1-eso-10.1177_23969873251315362 for The 6-months follow-up of the TREAT-CAD trial: Aspirin versus anticoagulation for stroke prevention in patients with cervical artery dissection by Stefan T Engelter, Lukas S Enz, Flavia Ravanelli, Josefin E Kaufmann, Henrik Gensicke, Sabine Schaedelin, Andreas R Luft, Christoph Globas, Barbara Goeggel-Simonetti, Urs Fischer, Davide Strambo, Georg Kägi, Krassen Nedeltchev, Timo Kahles, Lars Kellert, Sverre Rosenbaum, Regina von Rennenberg, Alex Brehm, David Seiffge, Susanne Renaud, Tobias Brandt, Hakan Sarikaya, Annaelle Zietz, Johannes Wischmann, Alexandros A Polymeris, Sandro Fischer, Leo H Bonati, Gian Marco De Marchis, Nils Peters, Christian H Nolte, Hanne Christensen, Susanne Wegener, Marios-Nikos Psychogios, Marcel Arnold, Philippe Lyrer and Christopher Traenka in European Stroke Journal
