# Supplementary material for: The 6-months follow-up of the TREAT-CAD trial: Aspirin versus anticoagulation for stroke prevention in patients with cervical artery dissection
Source: Eur Stroke J. 2025 Feb 5;10(3):871–81. doi: 10.1177/23969873251315362 (PMC11803590; doi:10.1177/23969873251315362)
Supplement: sj-docx-1-eso-10.1177_23969873251315362 – Supplemental material for The 6-months follow-up of the TREAT-CAD trial: Aspirin versus anticoagulation for stroke prevention in patients with cervical artery dissection [file sj-docx-1-eso-10.1177_23969873251315362.docx]

Supplements

**Participating centres and their numbers of recruited participants with 6-month follow-up (n=127) in alphabetical order:**

**Switzerland:**

Aarau (n=5): Cantonal Hospital Aarau, Aarau; Switzerland

Basel (n=58): University Hospital Basel; Switzerland

Bern (n=17): University Hospital, Bern; Switzerland

St. Gallen (n=6): Kantonsspital St. Gallen; Switzerland

Zurich (n=23): University Hospital Zurich, Switzerland

**Germany:**

Berlin (n=10): Charité-Universitätsmedizin, Berlin; Germany

Munich (n=6): University Hospital, Ludwig-Maximilians University, Munich, Germany

**Denmark:**

Copenhagen (n=2): Bispebjerg Hospital & University of Copenhagen, Copenhagen, Denmark

**Detailed definition of outcomes**

The main composite outcome measure includes the following efficacy and safety outcome measures during the treatment period:

1. Cerebral Ischemic events (clinical) or surrogate findings for cerebral ischemia:

- occurrence of any ischemic stroke (definition includes ‘TIA’ with DWI lesions)
- new acute lesions on diffusion-weighted MRI (DWI)

2. Hemorrhages (clinical) or surrogate findings:

- any major extracranial hemorrhage (defined as any clinical apparent bleeding requiring any kind of intervention (including hospitalization or prolongation of hospitalization] or leading to death)
- any symptomatic intracranial hemorrhage (any documented intracranial hemorrhage that was temporally related to any deterioration in the patient’s clinical condition)
- any asymptomatic micro- or macrobleeds (visible on follow-up brain T2*-MRI (or SWI), which were absent on previous MR-scans)

3. Death (of any course).

**Figure e-1: MRI-outcomes**

| 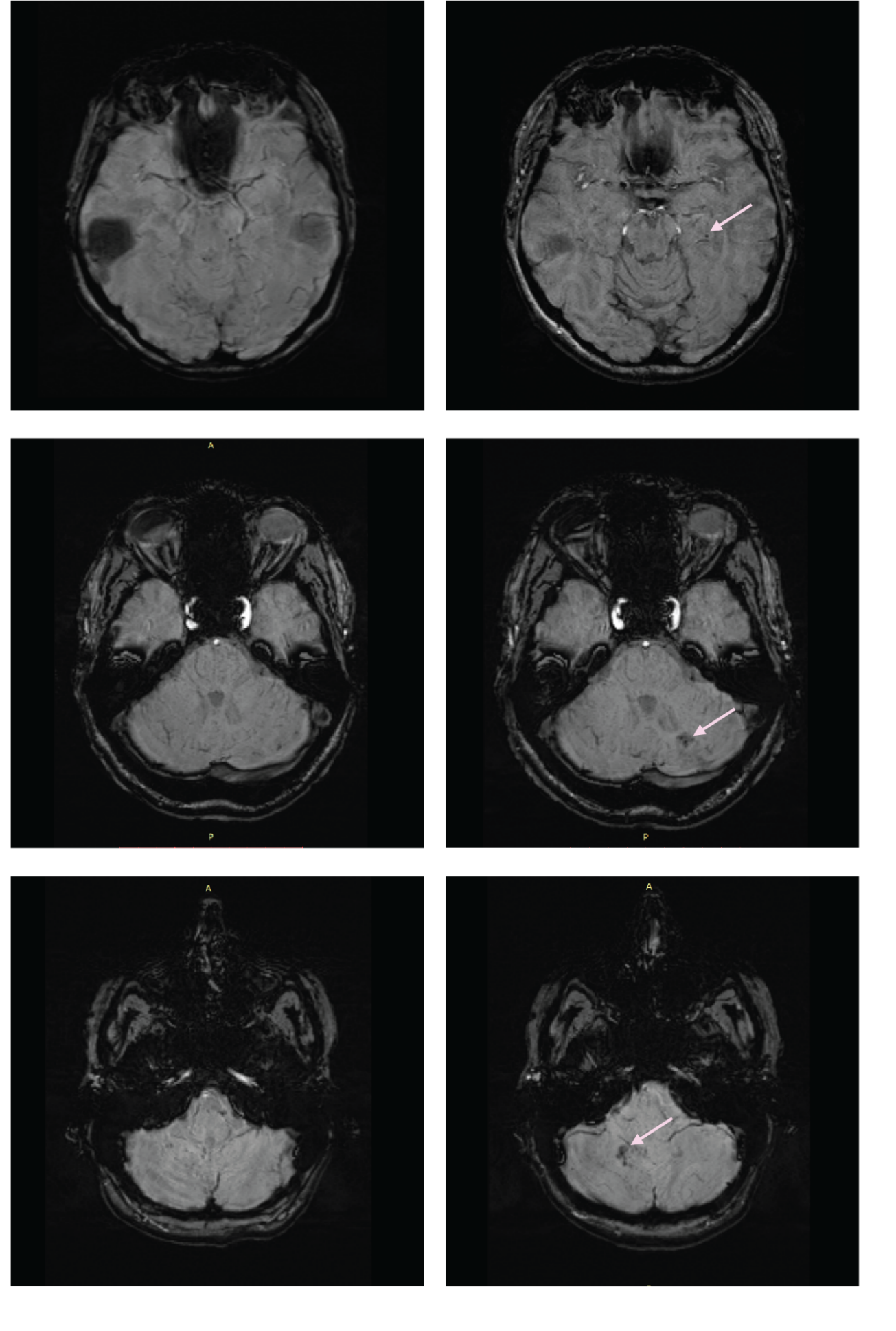 |
| --- |
| **Legend**. Susceptibility weighted MR-images (SWI) of the three participants with paramagnetic lesions visible newly visible at the 6-month-follow-up (right column, arrow) but not already at the follow-up MR-images at 14+/-7 days (left column). |

**Table e-1: Comparison of TREAT-CAD 3-6 months follow-up with CADISS and STOP-CAD follow-ups**

|  | TREAT-CAD | | CADISS^1^ | | STOP-CAD^2^ | |
| --- | --- | --- | --- | --- | --- | --- |
| Study Design | Randomized clinical trial 0-3 months with an extended open-label follow-up 3-6 months | | Randomized controlled  clinical trial | | Retrospective observational  study | |
| Analysis | Per protocol  As-treated | | Per Protocol | | - | |
| Time window | 0-3 months after randomization  3-6 months after randomization | | 0-3 months after randomization  3-12 months after randomization | | 0-3 months after randomization  3-6 months after dissection | |
| Outcome type | Aspirin | Anticoagulants | Antiplatelets | Anticoagulants | Antiplatelets | Anticoagulants |
| Primary endpoints total | 21/91 (23.1%)  3/93 (3.2%) | 12/82 (14.6%)  1/29 (3.4%) | 5/101 (5.0%)  2/101 (2.0%) | 5/96 (5.2%)  1/96 (1.0%) | 137/2453 (5.6%)  9/1790 (0.5%)** | 35/402 (8.7%)  9/370.5  (2.4%)** |
| *Components of the primary endpoint* |  |  |  |  |  |  |
| Clinical outcomes (all) | 7/91 (7.7%)  1/93 (1.1%) | 1/82 (1.2%)  0/29 | 5/101 (5.0%)  2/101 (2.0%) | 5/96 (5.2%)  1/96 (1.0%) | 137/2453 (5.6%)  9/1790 (0.5%)** | 35/402 (8.7%)  9/370.5  (2.4%)** |
| - Ischemic stroke† | 7/91 (7.7%)  0/93 | 0/82  0/29 | 5/101 (5.0%)  1/101 (1.0%) | 4/96 (4.2%)  1/96 (1.0%) | 121/2453 (4.9%)  9/1767 (0.5%)** | 29/402 (7.2%)  3/368  (0.8%)** |
| - Major extracranial hemorrhage | 0/91  1/93 (1.1%) | 1 (1.2%)  0/29 | 0/101  0/101 | 0/96  0/96 | 16/2453 (0.7%)  0/1813** | 6/402 (1.5%)  6/373  (1.6%)** |
| - Symptomatic intracranial hemorrhage | 0/91  0/93 | 0/82  0/29 | 0/101  0/101 | 1/96 (1.0%)  0/96 |  |  |
| - Death | 0/91  0/93 | 0/82  0/29 | 0/101  1/101 (1.0%) | 0/96  0/96 | Not assessed | |
| MRI-outcomes (all) | 20 (21.9%)  2/93 (2.2%) | 11/82(13.4%)  1/29 (3.4%) | Not assessed | | Not assessed | |

**Legend**. † Including TIA in the TREAT-CAD and CADISS trial but not STOP-CAD. In STOP-CAD, this also included new or enlarged ischemic stroke in the territory of the dissected artery only visible in MRI but with no clinical worsening. *The number of patients available depended on the outcome. We chose the number of patients at risk at 90 days for the denominator and the mean number at risk for both outcomes as the denominator for the summary of all outcomes. ** Outcomes were not explicitly reported for 3 to 6 months, the numbers here were estimated from Figure 2 of the STOP-CAD publication (survival curves). This may not be exact.

**References**

1. Markus HS, Levi C, King A, et al. Antiplatelet Therapy vs Anticoagulation Therapy in Cervical Artery Dissection: The Cervical Artery Dissection in Stroke Study (CADISS) Randomized Clinical Trial Final Results. JAMA Neurol 2019; 76: 657-664. 2019/02/26. DOI: 10.1001/jamaneurol.2019.0072.

2. Yaghi S, Shu L, Mandel DM, et al. Antithrombotic Treatment for Stroke Prevention in Cervical Artery Dissection: The STOP-CAD Study. Stroke 2024 20240209. DOI: 10.1161/strokeaha.123.045731.
